# Comparison of the Airtraq laryngoscope and the GlideScope for double-lumen tube intubation in patients with predicted normal airways: a prospective randomized trial

**DOI:** 10.1186/s12871-015-0037-5

**Published:** 2015-04-28

**Authors:** Jie Yi, Yahong Gong, Xiang Quan, Yuguang Huang

**Affiliations:** Department of Anesthesiology, Chinese Academy of Medical Science, Peking Union Medical College Hospital, No.1 Shuaifuyuan Wangfujing Street, Beijing, 100730 P.R. China

**Keywords:** Airtraq laryngoscope, Glidescope, Double-lumen tube, Intubation, Airway

## Abstract

**Background:**

The Airtraq laryngoscope and the GlideScope are commonly used in many airway scenarios. However, their features have not been fully described for double-lumen tube intubation. A prospective randomized study was designed to compare their intubation performances in thoracic surgery patients.

**Methods:**

Seventy ASA physical status I and II patients with predicted normal airway were scheduled for thoracic surgeries with double-lumen tube intubation. They were randomly assigned to one of two groups and intubated with either the Airtraq laryngoscope (group A, n = 35) or the GlideScope (group G, n = 35). Airway assessments were performed prior to anesthesia, and all patients were induced with a standard anesthetic regimen. The Cormack-Lehane grades were initially evaluated with a Macintosh laryngoscope and subsequently with the group-specific laryngoscope before intubation. Intubation time was recorded as the primary outcome. The Cormack-Lehane grade, the success of the first intubation attempt, the intubation difficulty scales and ease of tube advancement were noted. Hemodynamic variables during intubation and incidence of post-operative sore throat were documented as well.

**Results:**

The intubation time of group A was shorter than that of group G (36.6 ± 20.2 s vs. 54.6 ± 25.7 s, p = 0.002). The Cormack-Lehane grade (I/II/III/IV) was significantly better in group A (33/2/0/0 *vs.* 28/7/0/0, p = 0.042). The mean arterial pressure and heart rate rose to higher levels during intubation with the GlideScope than with the Airtraq laryngoscope. The success of the first intubation attempt and the intubation difficulty scales were comparable between the two groups. The numbers of patients who experienced postoperative sore throat were similar (6 *vs.* 8) in the two groups.

**Conclusions:**

Compared with the GlideScope, the specially designed Airtraq laryngoscope might be more suitable for double-lumen tube intubations in patients with predicted normal airway.

**Trial registration:**

www.chictr.org Identifier: ChiCTR-TRC-11001628

## Background

Many thoracic surgeries require one-lung ventilation for better surgical vision. Double-lumen tube intubation is commonly applied to achieve one-lung ventilation [1]. However, such intubation can occasionally be difficult, particularly in difficult airway cases, due to the larger size and complex shape of the double-lumen tube.

The American Society of Anesthesiologists Difficult Airway Algorithm was updated in 2013. Video-assisted laryngoscopy is recommended as an initial approach during intubation [2]. The Airtraq laryngoscope (Prodol Meditec S.A., Vizcaya, Spain) and the GlideScope (Verathon Inc., Bothell, WA, USA) were introduced in recent years. Their common features include specially designed blades and the video cameras, integrated into the GlideScope blade or as external accessory attached onto the Airtraq Laryngoscope, which provide better laryngeal views [3,4]. However, the Airtraq Laryngoscope has a side channel to guide endotracheal tube advancement which is different from the GlideScope. Once the vocal cord has been optimally exposed, the endotracheal tube is introduced into the tracheal through the side channel.

Literature has demonstrated that the Airtraq Laryngoscope and the GlideScope can facilitate tracheal intubation in normal and difficult airways both in manikins and patients [4-8]. However, compared with single lumen tube intubation, few studies have specifically focused on double-lumen tube intubation with video laryngoscopes [9-12]. Recently, several studies have compared video laryngoscopes to the conventional Macintosh laryngoscope for double-lumen tube intubations [13-15]. The results were inconsistent in comparison of the GlideScope and Macintosh laryngoscope and the advantages of the Airtraq laryngoscope were not apparent over the Macintosh laryngoscope. Furthermore, the differences between video laryngoscopes in terms of intubation efficacy and performance have not been fully revealed so far. We designed a prospective randomized study to compare the Airtraq laryngoscope and the GlideScope for double-lumen tube intubation. Our hypothesis was that the specially designed Airtraq laryngoscope would likely be superior to the GlideScope for double-lumen tube intubation.

## Methods

This study was registered at www.chictr.org (identifier: ChiCTR-TRC-11001628). The protocol was approved by the Ethics Committee of Peking Union Medical College Hospital, Beijing, China (chairperson: Professor Jie Chen) on the 20th of July, 2011 (Ethical Committee approval No. S-384). Written informed consent was obtained from all patients.

Seventy patients with ASA physical status I-II and aged between 18–75 years were enrolled in this study. The patients were scheduled for thoracic surgeries including thoracotomy or video-assisted thoracoscopic surgeries with double-lumen tube intubations. The exclusion criteria included emergency thoracic surgeries, histories of previous failed or difficult intubation or identified oral cavity or tracheal masses. Patients were also excluded from the study if they presented more than two of the following risks: mouth openings < 3 cm, thyromental distance <6 cm, Mallampati class III or IV, neck flexation and extension <30° [16].

The patients were randomly assigned to one of two groups, group A (intubation with the Airtraq laryngoscope, n = 35) or group G (intubation with the GlideScope, n = 35), based on computer-generated random numbers that were sealed in an envelope and disclosed prior to general anesthesia. Airway assessments, including mouth opening, thyromental distance (TMD), Mallampati grade, atlanto-occipital joint movement (A-OJM) and the upper lip bite test (ULBT), were performed before the induction of anesthesia [17].

Non-invasive blood pressure, electrocardiogram and pulse oximetry were normally monitored for each patient. General anesthesia was induced with a standardized regimen that included intravenous fentanyl (2 μg/kg) and propofol (2.5 mg/kg). When the patient lost consciousness, rocuronium (1 mg/kg) was administered. A peripheral nerve stimulator (Multistim VARIO, Pajunk® GmbH, Geisingen, Germany) was used to confirm that the train-of-four ratio decreased to zero, which indicated an ideal intubation condition had been achieved. Mask ventilation with 100% oxygen was delivered to the patients during induction. Prior to intubation, the glottis exposure was assessed twice according to the Cormack-Lehane grade [18], initially with a Macintosh laryngoscope and then with the corresponding laryngoscope in each assigned group, followed by intubation. Left or right sided of double lumen tube (Broncho-Cath^™^ Tyco Healthcare Mallinckrodt Medical, Athone Ireland) was chosen according to surgeon’s request. 35 F or 37 F tube was selected for female, and 37 F or 39 F tube for male. In group G, the double lumen tube was shaped with its own stylet inside the tube to fit along the curvature of the blade. When the blue cuff of the bronchial lumen passed through the vocal cords, the stylet was removed gradually, and the tube was rotated counterclockwise to enter the trachea according to the suggestions of Bustamante et al. [12]. For the Airtraq laryngoscope intubation, the original stylet inside the tube was removed, and the tube was preloaded into the conduit of the blade before intubation as recommended by the manufacturer. Once the tip of bronchial lumen passed through the vocal cords, advancement was halted, and the tube was tightly secured before removal of the Airtraq laryngoscope from the mouth. The tube was then advanced further to enter the bronchus. All intubations were performed by a single senior anesthesiologist (J. Yi) with experience in more than 30 double-lumen tube intubation cases with the Airtraq laryngoscope and the GlideScope.

As the primary outcome, intubation time was recorded by an independent staff member who was unaware of the study protocol. The intubation time was defined as the time period between the laryngoscopes passed the patient’s lips and the completion of the tube advancement into the trachea. Secondary outcomes were evaluated for all patients. They included the success of the first intubation attempt, the intubation difficulty scale (IDS) as described by Adnet et al. [19], and the ease of insertion of the laryngoscope and tube advancement, which were subjectively rated from 0 to 3 (0, very easy; 1, easy; 2 difficult; and 3, very difficult) by the intubating anesthesiologist. The mean arterial pressure and heart rate were recorded prior to intubation as baseline values, and these measures were repeated at the time of intubation completion and 3 min after intubation. The incidence of post-operative sore throat was assessed within 24 h of the surgery.

Tube placement was confirmed by capnography, and further correction of the bronchial lumen in the bronchus was performed with flexible fiberoptic bronchoscopy. If the pulse oximetry dropped below 92% during the first intubation attempt, mask ventilation was given to the patient until the SPO_2_ returned to 100%. Then, the same laryngoscope was used in a second attempt. If intubation failed again, the patient was awakened and intubated via fiberoptic bronchoscopy.

The sample size was estimated based on the intubation time with a type I error of 0.05 and a power of 80%. Assuming a possible difference in mean intubation time of 10 s and a common standard deviation of 12 s in each of the groups based on our pilot data and other publications [13-15], a sample size of 30 patients in each group would be necessary. To allow for missing cases and dropouts due to various reasons, we recruited a minimum of 35 patients for each group in this study. Continuous data are presented as means ± SDs and were analyzed with one-way ANOVAs followed by the post hoc test of Least Significant Difference (LSD). The intra- and inter-groups glottis exposures, based on Cormack-Lehane grade, were analyzed by Kappa test and Mann–Whitney U test, respectively. Success of the first attempt and the incidence of sore throat were analyzed using chi-squared tests, and other ordinal data were analyzed with Mann–Whitney U tests. The hemodynamic changes were analyzed with repeated-measures ANOVA. Significance was accepted at P < 0.05. All statistical analyses were performed using SPSS 18.0 (SPSS, Chicago, IL USA).

## Results

**Figure 1:**
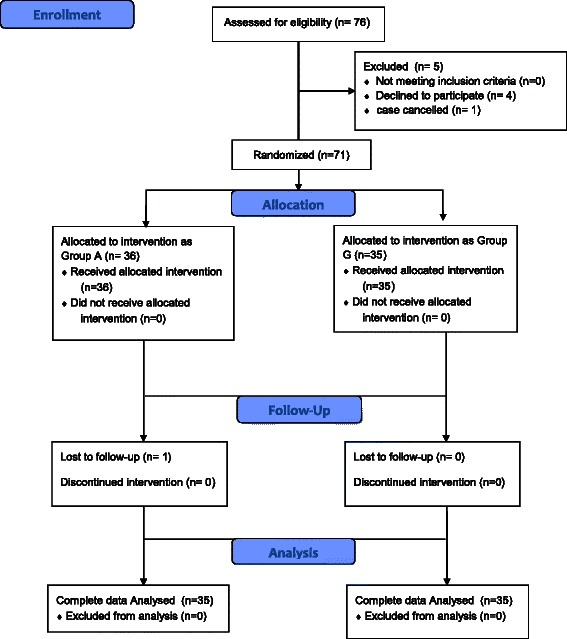
CONSORT of flow diagram.

The study began on Nov 17th, 2011 and ended on May 24th, 2012. 76 patients in total were assessed initially for eligibility during study period. One case was cancelled, four patients refused to sign the study consent form and one missed follow-up postoperatively. Finally 70 patients were enrolled in the study analysis (Figure 1). There were no significant differences between two groups in terms of the demographic data or airway assessments (Table 1).

**Table 1:** **Demographic data and airway assessments**

	Group A (n = 35)	Group G (n = 35)	P-value
Age (years)	56 ± 15 (19–75)	55 ± 13 (24–75)	0.772
Gender (male/female, n)	22/13	21/14	0.811
Weight (Kg)	64.6 ± 12.2 (44–87)	65.3 ± 10.8 (46–88)	0.76
Height (cm)	167 ± 8 (154–180)	165 ± 8 (148–180)	0.483
Mouth opening (cm)	4.4 ± 0.6 (3.5-6)	4.3 ± 0.5 (3.5-6)	0.425
TMD (cm)	7.5 ± 1.1 (5–9.5)	7.2 ± 1.0 (5–10)	0.316
Mallampati classification (I/II/III/IV, n)	17/13/5/0	18/14/3/0	0.307
A-OJM (>30°, n)	34	35	0.314
ULBT (A/B/C, n)	27/7/1	22/13/0	0.064

Data are presented as means ± standard deviations (range) or the numbers of patients. Group A: the patients were intubated with the Airtraq laryngoscope; Group G: the patients were intubated with the GlideScope. TMD = thyromental distance; A-OJM = atlanto-occipital joint movement; ULBT = upper lip bite test, A: lower incisors can bite the upper lip above the vermilion line; B: lower incisors can bite the upper lip below the vermillion line; C: lower incisors cannot bite the upper lip [16].

**Figure 2:**
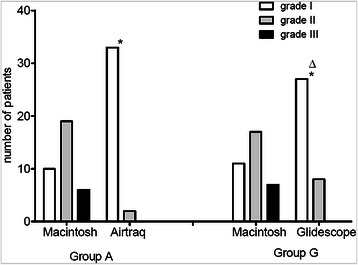
Comparison of the Cormack-Lehane grades of the two groups. group A: the patients were intubated with the Airtraq laryngoscope; group G: the patients were intubated with the GlideScope. Initial assessments of the laryngeal views (Cormack-Lehane grade I/II/III) were performed with the Macintosh laryngoscope (Macintosh) and subsequently with the Airtraq laryngoscope (Airtraq) or the GlideScope (GlideScope) in the corresponding groups. * significant compared with the initial Macintosh assessment within groups based on Kappa test (Kappa value = −0.53 in group A; −0.32 in group G); ^Δ^ significant difference in the assessments performed with the Airtraq laryngoscope and the GlideScope according to Mann–Whitney U test, P = 0.042 < 0.05.

All patients were successfully intubated with the corresponding laryngoscope. The intubation time with the Airtraq laryngoscope was shorter than that with the GlideScope (36.6 ± 20.2 s vs. 54.6 ± 25.7 s, P = 0.002 < 0.01). The initial glottis exposures with the Macintosh laryngoscope were comparable between the two groups. The second assessments with the Airtraq laryngoscope and the GlideScope were better than the initial grades based on the Cormack-Lehane grades (I/II/III: 10/19/6 vs. 33/2/0 and 11/17/7 vs. 27/8/0, respectively). Furthermore, the Airtraq laryngoscope provided better glottis views than did the GlideScope (P = 0.042 < 0.05, Figure 2).

The success of the first intubation attempt and the intubation difficulty scales (IDS) were similar between two devices. The distribution of IDS, either rated as “easy” (IDS = 0) or as “slight difficulty” (1 < IDS < 5), were comparable in two groups (Table 2). These two laryngoscopes were easy to insert in most patients. Only one patient in group A was rated as difficult insertion because of dental problem. 5 cases and 3 cases in group A were considered as difficult or very difficult to advance the tube. Similarly, 5 cases and one case were difficult or very difficult in group G as well in terms of tube advancement.

**Table 2:** **Intubation data of the two laryngoscopes**

	Group A (n = 35)	Group G (n = 35)	P-value
Intubation time (s)	36.6 ± 20.2 (12–91)	54.6 ± 25.7 (28–133)	P = 0.002*
Success rate of first intubation attempt (n, %)	33 (94%)	34 (97%)	0.55
Intubation difficulty scale (IDS) (0/1/2/3/4, n)	10/17/5/2/1	9/13/7/5/1	0.327
Ease of laryngoscope insertion (0/1/2/3, n)	33/1/1/0	33/2/0/0	0.98
Ease of tube advancement (0/1/2/3, n)	24/3/5/3	26/3/5/1	0.525
Sore throat (n, %)	6 (17%)	8 (23%)	0.766
DLT sizes (39 F/37 F/35 F, n)	15/12/8	12/12/11	0.378
DLT type (left/right sided)	23/12	28/7	0.182

Group A: the patients were intubated with the Airtraq laryngoscope; Group G: the patients were intubated with the GlideScope. IDS score (0/1/2/3/4) as explained by Adnet, et al. [18]. Ease of laryngoscope insertion and tube advancement (0 = very easy, 1 = easy, 2 = difficult, 3 = very difficult). Data are presented as mean ± standard deviations (ranges) or as the numbers of patients, * statistically significant difference between two groups.

**Figure 3:**
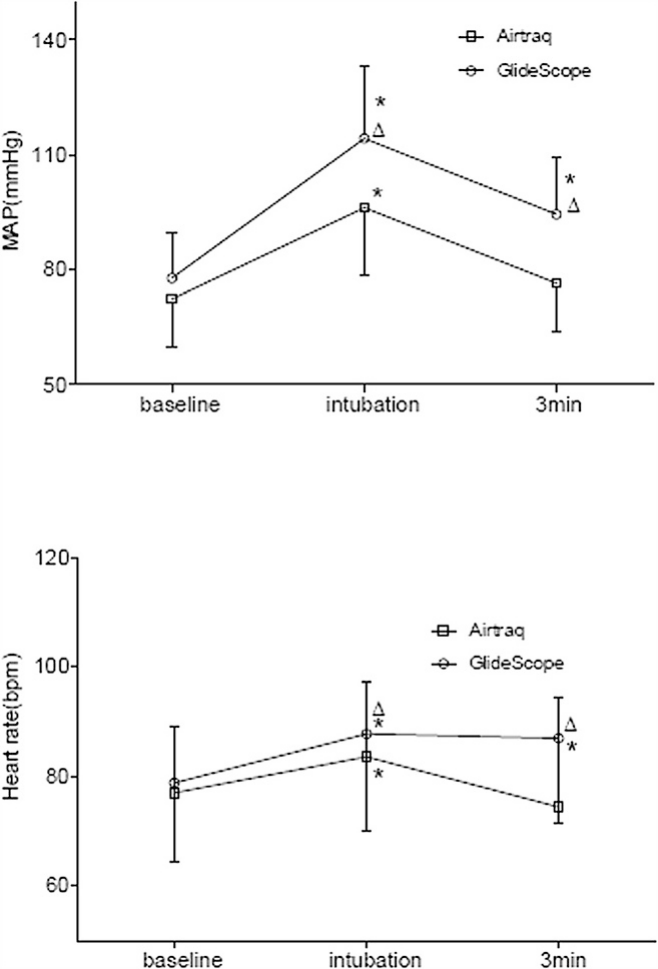
Hemodynamics changes during the intubation periods with the two laryngoscopes. Mean arterial pressure (MAP, upper) and heart rate (lower) are shown. The values increase at the time of intubation and return to baseline at 3 min after intubation with the exception of the GlideScope group. The data are presented as means ± SDs. Baseline: prior to intubation; Intubation: at the time of intubation; 3 min: 3 min after intubation. *P < 0.05, compared with baseline; ^Δ^P < 0.01, for the comparison between two groups.

The mean arterial pressures and heart rates increased during the period of intubation in both groups. Significant differences were found between two groups on the levels of increase in blood pressures and heart rates. In addition, both blood pressure and heart rate returned to baseline after intubation in group A, while in group G, they kept in higher levels 3 min after intubation (Figure 3). The incidences of sore throat were comparable between group A (6, 17%) and group G (8, 23%) after 24 h of postoperative followed-up.

## Discussion

Video laryngoscope, like the GlideScope and the Airtraq laryngoscope, has advantages of providing better glottis exposure and facilitating the intubation. However, the size and shape of double lumen tube can make intubation difficult and attenuate the advantages of video laryngoscopes. In this study, we found that all double lumen tube intubations were successful in patients with normal airway. However, compared with the GlideScope, the application of the Airtraq laryngoscope was associated with shorter intubation time, greater improvement of the Cormack-Lehane grade and less hemodynamic responses during intubation.

Intubation time was evaluated as the primary outcome because it is considered to be a comprehensive endpoint for the evaluation of intubation techniques and performances [20]. Small differences in intubation time, for example the difference of 18 s between the Airtraq laryngoscope and GlideScope observed in our study, might not have clinically significant impact but might reflect the ease of manipulation of different laryngoscopes. The Airtraq laryngoscope presented a shorter intubation time that is similar to that reported in Wasems’s study [13]. However, the reported time to intubation with the GlideScope have varied in different studies. Russell et al. [15] reported a median intubation time of 70 s, while Hsu et al. [14] reported a 45.6 s mean intubation time compared with that of the Macintosh laryngoscope. The diversity of these results might indicate difficulties in the manipulations of double-lumen tube intubation. Regarding the GlideScope, as Bustamante et al. suggested [12], the maneuvering of the tube is slightly complicated. Counterclockwise rotation was needed as the stylet was removed to facilitate tube passage into the vocal cords, and occasionally more rotation of the tube was needed to achieve further advancement [21]. All of the manipulations are time-consuming, which might lead to longer intubation duration. In contrast, the Airtraq laryngoscope has an integrated tube conduit through which the double-lumen tube can easily be preloaded and inserted along the conduit into the vocal cords. This difference might explain shorter intubation time of the Airtraq group, and this notion is also supported by the results of Savoldelli’s study [22].

One of the advantages of video laryngoscopes is that they provide better glottic views than do conventional Macintosh laryngoscopes [3-7]. Some literatures have introduced the percentage of glottic opening (POGO) as another measure of glottic view and showed that it has good intra- and inter-observer reliability [23-25]. In the present study, the glottic view was evaluated based on the Cormack-Lehane grades which is very familiar to anesthesiologists for assessing the laryngeal view, in particular with the Macintosh laryngoscope. We used the Macintosh laryngoscope to assess the initial view of glottis in order to compare intra- and inter-groups. Indeed, it has shown similar results in that the glottic views were improved with both the Airtraq laryngoscope and the GlideScope compared with the initial exposures with the Macintosh laryngoscope. Additionally, the distribution of the Cormack-Lehane grades for the Airtraq laryngoscope was better than that for the GlideScope. Several studies have also demonstrated that the GlideScope is less effective than the Airtraq laryngoscope in improving glottic view in both normal and difficult airway scenarios [8,21]. Although large improvements in laryngeal exposure might not have significant clinical affects, these characteristics support the relative advantage of the use of the Airtraq laryngoscope for double-lumen tube intubation.

Literatures demonstrated that an improved glottis view may not be associated directly with easy intubation, in particular with the Glidescope [26,27]. The potential explanation may focus on the intubation under indirect view via a monitor requiring complex manipulation of the tube. Intubation difficulty scores (IDSs) are typically used to indicate the difficulties of intubations with different laryngoscopes [19,28,29], although it remains controversial whether the IDS is suitable for the evaluation of indirect laryngoscopes [30]. In the present study, the distributions of IDS scores were similar between the two groups, and no patients had IDS scores of 5 or more. These findings might indicate that double-lumen tube intubation using either of these two laryngoscopes is not difficult. Additionally, the distributions of the ease of laryngoscope insertion and tube advancement, as subjectively assessed by the intubator, were comparable between the two groups. Twenty seven patients in group A and 29 patients in group G were given intubation manipulation ratings of very easy or easy. These findings in the study might suggest that the Airtraq laryngoscope and the GlideScope have equivalent manipulation difficulties in double-lumen tube intubations despite the acknowledged limitations of the subjective endpoints.

In the present study, the incidences of postoperative sore throat in group A and group G were 17% and 23%, respectively. Wasem reported that 24% of patients complain of sore throat postoperatively following intubation of double lumen tube with the Airtraq laryngoscope [13]. Hsu et al. [14] reported that the use of the GlideScope is associated with a 20% incidence of sore throat, and these authors also used technique described by Bustamante et al. [12]. However, Russell et al. [15] reported that the postoperative voice changes occurred in 58% of the patients in their GlideScope group, and these authors did not use the maneuver of Bustamante [12]. Indeed, manipulations in the pharyngolaryngeal space might play important roles in the occurrence of sore throat [21,31,32]. Additionally, the large size and curvature of double lumen tubes likely cause intubation difficulty and increases intubation complications.

The mean arterial pressure and heart rate increased as predicted during the intubations. However, in contrast to group A, the blood pressures and heart rates of the patients in group G reached higher levels and did not return to baseline 3 min after intubation. These results suggest that intubation with the GlideScope might cause stronger sympathetic effects. We found that longer intubation times and complicated tube manipulations were associated with GlideScope intubation. Huang et al. [33] demonstrated that longer intubation time is responsible for greater hemodynamic responses. Moreover, Takahashi et al. [34] concluded that the tracheal stimulation caused by endotracheal tube manipulation is the major determinant of hemodynamic changes. These characteristics of the GlideScope might help to explain the difference in hemodynamic responses between the two groups.

There are several limitations to our study. First, these two laryngoscopes are completely different in terms of shape and size; thus, it was impossible to blind the investigator in this study. Second, the patients in the study were predicted to have normal airways; thus, any differences between the uses of these video laryngoscopes for double-lumen tube intubation of patients with difficult airways could not be detected. Further investigations are needed to demonstrate the properties of these video laryngoscopes during difficult intubations. Finally, similar to the study by Wasem et al. [13], all of the intubation manipulations were performed by a single anesthesiologist. Hsu et al. performed an investigation that involved two anesthesiologists, and Russell et al. enrolled 30 anesthesiologists in their study [14,15]. The use of a sole intubator versus many intubators poses a dilemma. The influence of the learning curve for the use of the device should be taken into account if one or two intubators are involved in a study. For many anesthesiologists, the diversity of their intubation abilities across different laryngoscopes might cause even larger biases in the results.

## Conclusions

In conclusion, the Airtraq laryngoscope and the GlideScope are safe and effective alternative devices for double lumen tube intubation. For patients with predicted normal airways, the specially designed Airtraq laryngoscope was found to be advantageous compared with the GlideScope. However, further studies are needed to demonstrate the benefits of the devices in different clinical settings.

## Abbreviations

TMD: Thyromental distance
A-OJM: Atlanto-occipital joint movement
ULBT: The upper lip bite test
IDS: Intubation difficulty scale
